# Enhancing tobacco quality through microbial-enzymatic processing: mechanisms, applications, and future perspectives

**DOI:** 10.3389/fbioe.2026.1895350

**Published:** 2026-07-16

**Authors:** Lei Xu, Peng Zhang, Hui Zhang, Ping-Ping Liu, Zhe Jin, Jin-Song Gong, Zhen-Ming Lu, Qing-Xia Zheng, Guo-Yun Xu, Niu Zhai, Hui-Na Zhou, Yu-E. Li, Li-Feng Jin

**Affiliations:** 1 Zhengzhou Tobacco Research Institute of CNTC, Zhengzhou, China; 2 National Key Laboratory for Development and Utilization of Forest Food Resources, Zhejiang A&F University, Hangzhou, China; 3 China Tobacco Jilin Industrial Co., Ltd., Changchun, China; 4 School of Life Sciences and Health Engineering, Jiangnan University, Wuxi, China; 5 National Engineering Research Center for Cereal Fermentation and Food Biomanufacturing, School of Biotechnology, Jiangnan University, Wuxi, China

**Keywords:** aging process, enzymatic treatment, fermentation, microbial community, tobacco processing

## Abstract

Aging represents a critical stage in tobacco processing that balances the internal chemical composition of tobacco leaves, reduces undesirable odors and irritation, and enhances smoking quality. Microbial fermentation and enzymatic catalysis are pivotal to this process, as they not only accelerate the fermentation period but also regulate the internal chemical constituents of the leaves, thereby improving aging efficiency. Consequently, the screening of suitable enzymes and functional strains is of paramount importance. This review summarizes recent advances in the application of enzymes and microorganisms for tobacco quality improvement, aiming to elucidate the mechanisms of enzymatic catalysis degrades macromolecules and generates aroma compounds, while also revealing the metabolic regulatory functions of microbial communities. This review aims to provide researchers with a comprehensive understanding of the latest developments in this field, while offering insights into the mining and engineering of core enzyme systems and functional strains.

## 1 Introduction

Cigarette products are predominantly manufactured from tobacco (*Nicotiana tabacum* L.), a herbaceous plant of the Solanaceae family and an economically important crop worldwide (Ning et al., 2023a). However, freshly harvested or insufficiently processed tobacco leaves generally exhibit several undesirable quality attributes, including strong irritation, harsh smoke, insufficient aroma, excessive off-odors, and relatively high levels of risk-associated constituents (Li et al., 2020). These intrinsic limitations restrict their direct use in cigarette manufacturing and necessitate post-harvest processing strategies to improve sensory quality, chemical balance, and product stability. Among these strategies, fermentation and aging are regarded as essential processing steps, as they promote the gradual transformation of endogenous chemical constituents and contribute to the formation of a more desirable tobacco flavor profile (Ye et al., 2017; Mai et al., 2025; Yu et al., 2025).

Tobacco aging is not a simple storage process but a complex and dynamic biotransformation system involving intertwined physical, chemical, enzymatic, and microbial processes. During aging, major tobacco constituents, including carbohydrates, proteins, lipids, cellulose, pectin, carotenoids, polyphenols, and nitrogen-containing compounds, undergo progressive degradation, oxidation, hydrolysis, condensation, and secondary conversion (Zhou et al., 2020). These reactions reshape the chemical matrix of tobacco leaves and influence the formation of aroma-active compounds, taste-related metabolites, and irritation-associated substances. For example, protein hydrolysis can release free amino acids and small peptides, which may further participate in Maillard-type reactions and contribute to flavor development. Carotenoid degradation generates norisoprenoid aroma compounds, whereas lipid oxidation and fatty acid metabolism provide aldehydes, ketones, alcohols, and esters that are closely associated with tobacco aroma characteristics. Therefore, the quality improvement of aged tobacco depends largely on the coordinated regulation of multiple biochemical pathways rather than on the change of a single chemical component. Microorganisms and enzymes are central biocatalytic drivers of these transformations (Zhu et al., 2015; Wang et al., 2018). Indigenous or exogenously introduced microbial communities can secrete diverse enzymes, including proteases, amylases, cellulases, pectinases, lipases, oxidoreductases, and glycosidases, thereby accelerating the degradation of macromolecules and promoting the generation of flavor-related metabolites (Zhu et al., 2015; Banožić et al., 2020; Dai et al., 2020). In parallel, endogenous tobacco enzymes and exogenous enzyme preparations can participate in the selective conversion of specific substrates, such as proteins, starch, cellulose, carotenoids, phenolic compounds, and alkaloids. Through these catalytic processes, microbial and enzymatic systems may improve aroma richness, reduce harshness, decrease undesirable odors, modulate nicotine and other harmful constituents, and inhibit mold growth during storage or fermentation (Ning et al., 2023b; Fan et al., 2023). Thus, biocatalysis provides an important theoretical and technological basis for controllable tobacco aging and quality improvement.

In recent years, increasing attention has been paid to the targeted use of functional microorganisms and enzyme preparations in tobacco processing. For instance, specific fungal or bacterial strains have been reported to improve tobacco flavor by producing lipoxygenase, peroxidase, protease, cellulase, or other functional enzymes. Exogenous enzymes, such as neutral protease, amylase, cellulase, and pectinase, have also been applied to regulate the contents of sugars, amino acids, organic acids, nitrogenous compounds, and aroma precursors. These studies indicate that microbial and enzymatic interventions can shorten aging time and improve sensory quality. However, most existing studies remain focused on isolated strains, single enzyme systems, or endpoint chemical indicators. The dynamic interactions among microbial succession, enzyme expression, substrate transformation, and sensory quality formation are still insufficiently understood. In particular, the causal relationships between specific microbial taxa, key enzyme activities, metabolic pathways, and tobacco quality traits have not been systematically clarified. This limitation has become a major bottleneck in the development of precise and controllable biocatalytic strategies for tobacco processing. Tobacco leaves represent a heterogeneous solid-state matrix characterized by low water activity, complex chemical composition, spatially uneven microbial distribution, and dynamic environmental conditions. These features make it difficult to determine which microorganisms are metabolically active, which enzymes are functionally dominant, and which biochemical reactions are directly responsible for desirable quality changes. Moreover, conventional culture-dependent methods and bulk chemical analyses often fail to capture the temporal, spatial, and functional heterogeneity of microbial and enzymatic processes. Therefore, future research needs to move beyond descriptive correlations and establish mechanistic frameworks that integrate microbial ecology, enzymology, metabolomics, sensory evaluation, and process engineering.

In this context, this review systematically summarizes recent advances in biocatalysis for tobacco processing, with particular emphasis on microbial communities, key enzyme systems, substrate transformation pathways, and their effects on tobacco quality. First, we discuss the biochemical basis of tobacco aging and the major chemical constituents affected by microbial and enzymatic catalysis. Second, we review the roles of functional microorganisms and enzyme systems in regulating aroma formation, macromolecule degradation, harmful constituent reduction, and sensory quality improvement. Third, we critically evaluate current biotechnological strategies, including functional strain screening, exogenous enzyme application, microbial consortium construction, enzyme preparation development, and intelligent process regulation. Finally, we highlight future perspectives for the field, including mechanism-guided strain and enzyme mining, multi-omics-based functional verification, synthetic microbial communities, and precision biocatalysis for controllable tobacco aging.

## 2 Microbial-enzymatic applications in tobacco processing

Tobacco, as a complex organic matrix, contains diverse macromolecules including starch, cellulose, hemicellulose, pectin, and proteins. These constituents exist in specific structural configurations that not only influence the physicochemical properties of tobacco but also largely determine its quality and processing characteristics (Chen et al., 2021; Wen et al., 2022). During aging, these macromolecules undergo continuous degradation and transformation, resulting in dynamic shifts in total sugar and nitrogen contents. Such biochemical conversions facilitate various aromatic compounds, mitigates quality defects, and establishes unique flavor profiles (Wang et al., 2018; Chen et al., 2019).

### 2.1 Chemical basis for biocatalytic modification

**FIGURE 1:**
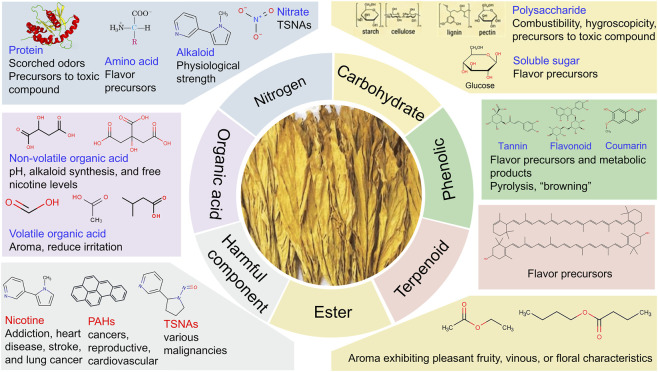
Primary chemical composition in tobacco.

Tobacco quality is fundamentally determined by its complex chemical matrix, which undergoes dynamic transformations during aging. These constituents can be broadly categorized into macromolecular precursors (e.g., proteins, polysaccharides) that require degradation to reduce irritation and improve combustibility, and small-molecular compounds (e.g., sugars, alkaloids, aroma precursors) that directly define sensory attributes such as sweetness, strength, and aromatic complexity. Furthermore, the balance between beneficial flavor components and hazardous substances generated during combustion is critical for both product acceptability and safety (Figure 1).

#### 2.1.1 Nitrogen compounds

Tobacco harbors a diverse array of nitrogen compounds, such as proteins, amino acids, alkaloids, nitrates, accounting for approximately 15% of the dry weight of tobacco leaf (Yu et al., 2025). These constituents serve as core chemical determinants of both tobacco quality and safety. Proteins, the primary macromolecular nitrogenous substances in tobacco, exhibit contents closely correlated with leaf maturity and aging processes. Although moderate protein levels contribute to smoke fullness, they also act as precursors to various toxic compounds (Gao et al., 2014; Mýtinová et al., 2011). During combustion, insufficiently degraded proteins undergo thermal pyrolysis, generating unpleasant scorched odors reminiscent of burning feathers, resulting in a bitter, pungent smoke that significantly increases irritation (Wu et al., 2021).

Amino acids, the fundamental building blocks of proteins, play a pivotal role in modulating tobacco flavor and aroma (Kanzler et al., 2017). Tobacco contains over 30 different types of amino acids that serve as key aroma precursors. Through the Maillard reaction with reducing sugars, these amino acids generate a spectrum of critical aroma compounds, such as pyrazines, pyrroles, and furans characterized by roasted, nutty, and baked notes (Chen et al., 2019). This process substantially enhances the complexity and refinement of the smoke profile.

Tobacco alkaloids are characteristic compounds unique to the plant, with their levels directly determining the physiological strength and satisfaction of the smoke, thereby forming the primary basis for the smoker’s psychological gratification (Shi et al., 2018). Nicotine, the predominant alkaloid, constitutes over 95% of total tobacco alkaloids (Zhong et al., 2010). During combustion, nicotine pyrolyzes to form aroma components like pyridine, contributing significantly to tobacco’s characteristic flavor. However, excessive level of nicotine can lead to a coarse smoke profile and heightened irritation, compromising smoking comfort (Weeks et al., 1995). Beyond these components, other nitrogen sources such as nitrates are non-negligible constituents of tobacco leaves. While nitrates contribute little directly to the sensory profile of the smoke, their levels critically affect the combustion rate and burning status of the leaf (Jiang F. et al., 2024; Tiecher et al., 2023).

#### 2.1.2 Carbohydrate

Tobacco leaves contain approximately 50% carbohydrates, comprising 6%–15% cellulose, 10%–15% pectin, 10%–30% starch, approximately 2% lignin, and varying amounts of sugars (Banožić et al., 2020; Reid et al., 1937). Depending on their molecular structures and functions, these components exert distinct mechanisms influencing tobacco quality.

Tobacco polysaccharides, including starch, pectin, cellulose, and lignin, often serve as critical limiting factors governing tobacco combustion and smoking quality (Banožić et al., 2020). Although starch is a primary metabolite in tobacco, insufficient degradation during leaf aging results in excessive residual levels, which impair combustibility, induce scorched and bitter off-notes, and severely compromise the color of cured leaves (Song et al., 2009). As major constituents of the cell wall, elevated levels of cellulose and lignin contribute to a coarse and pungent smoke profile. Specifically, lignin combustion generates phenolic compounds such as catechol, leading to astringency, dryness, distinct green off-notes, and throat irritation (Wang et al., 2025). Pectin, a hydrophilic colloid, directly dictates the physical properties of tobacco leaves; excessive content enhances hygroscopicity, predisposing leaves to mold or fragmentation, thereby detrimentally affecting processing quality and smoke taste (Zhu et al., 2014; Baliga et al., 2003). Furthermore, the combustion of macromolecular carbohydrates like starch and cellulose facilitates the formation of hazardous constituents, including formaldehyde, acetaldehyde, and polycyclic aromatic hydrocarbons, thereby escalating safety risks associated with cigarette consumption (Torikaiu et al., 2005; Sanders et al., 2003).

On the other hand, low-molecular-weight carbohydrates (soluble sugars) represent harmonizers and aroma precursors in tobacco (Roemer et al., 2012). Sugars in tobacco leaves, particularly reducing sugars such as glucose and fructose, not only significantly improve the sweetness and mellowness of tobacco but also undergo caramelization during combustion, generating characteristic aroma compounds with caramel and roasted notes (Roemer et al., 2012; Bak et al., 2005). More critically, as other key substrates in the Maillard reaction, reducing sugars exert effects similar to those of amino acids (Zhu et al., 2016). During the aging process, the enzymatic hydrolysis of polysaccharides such as starch into reducing sugars (e.g., glucose and fructose) is a critical process for quality enhancement. Studies indicate that consumer preference for tobacco products correlates positively with sugar content, and tobaccos with high sugar levels (8%–30%) are usually preferred by tobacco producers and consumers (Talhout et al., 2006).

#### 2.1.3 Phenolic and terpenoid compounds

Phenolic and terpenoid compounds represent pivotal metabolic products and aroma precursors in tobacco, critically influencing leaf coloration and sensory quality. Tobacco phenolics primarily comprise tannins (e.g., chlorogenic and cryptochlorogenic), flavonoids (e.g., rutin), and coumarins (e.g., scopoletin). Among these, chlorogenic acid, rutin, and scopoletin are abundant and serve as key indicators of tobacco quality (Meng et al., 2022). Sensory-wise, elevated chlorogenic acid enhances smoothness and aftertaste while reducing irritation, whereas scopoletin content correlates negatively with aroma quality (Hao et al., 2022). As aroma precursors, phenolics undergo pyrolysis during combustion to generate heterocyclic aroma compounds such as pyrazines and pyridines, thereby enriching the aromatic profile (Meng et al., 2022). However, phenolics are also primary drivers of tobacco “browning,” and excessive concentrations may impart bitterness and irritation to the smoke (Zou et al., 2023).

Terpenoids represent another crucial class of aroma precursors, including carotenoids, neophytadiene, and non-pigment compounds; their degradation is central to forming characteristic tobacco aromas. Neophytadiene, the most abundant neutral terpenoid, facilitates the transport of other aroma components, significantly enhancing smoke mellowness and reducing irritation (Li et al., 2020). Carotenoids, the major pigmented terpenoids, degrade via oxidation during leaf aging and combustion to yield key aroma constituents like β-damascenone, ionones, and megastigmatrienones. These compounds impart floral, fruity, woody, and sweet notes, fundamentally elevating aroma quality (Song et al., 2010; Tsuchiya et al., 2021). Additionally, cembranoids degrade during fermentation to produce aromas such as solanone and geranylacetone, further improving sensory attributes (Yu et al., 2025). Through enzymatic and non-enzymatic degradation, phenolics and terpenoids provide a diverse array of characteristic aromas, constituting the material basis for the unique flavor profile of tobacco.

#### 2.1.4 Organic acids and ester compounds

Organic acids and esters constitute another critical class of low-molecular-weight flavor components in tobacco, playing a pivotal role in pH regulation, aroma enhancement, and overall quality improvement (Baker et al., 2004). Tobacco organic acids are categorized into volatile and non-volatile fractions. Non-volatile organic acids, such as malic, citric, and oxalic acids, are abundant in tobacco leaves. Despite their weak inherent aromatic properties, these acids can modulate leaf pH, alkaloid synthesis, and free nicotine levels, thereby imparting mellowness and improving aftertaste. However, excessive concentrations may result in a flat or astringent smoke profile (Hu et al., 2022). Conversely, volatile organic acids (e.g., formic, acetic, and isovaleric acids) and esters are transferred directly into the mainstream smoke during combustion, contributing immediately to aroma and reducing irritation. For instance, benzoic acid imparts an almond-like note, whereas α-methylbutyric, isovaleric, and β-methylvaleric acids provide herbal and fruity nuances (Wang et al., 2008). Esters, primarily formed via the esterification of fatty alcohols, terpenoid alcohols, fatty acids, and sterols, typically exhibit pleasant fruity, vinous, or floral characteristics. Examples include ethyl acetate (banana), ethyl valerate (sweet apple), and butyl butyrate (pineapple) (Xu et al., 2020; Lu et al., 2021). These compounds effectively mask off-notes while enhancing the sweetness and smoothness of the smoke (Liu et al., 2016). Consequently, the moderate accumulation of organic acids and esters is essential for optimizing tobacco quality.

#### 2.1.5 Harmful components

The presence and control of harmful chemical constituents are indispensable safety metrics for evaluating tobacco quality, directly impacting consumer health risks. These hazardous substances primarily originate from the pyrolysis and synthesis of endogenous precursors during combustion. Key concerns include nicotine, polycyclic aromatic hydrocarbons (PAHs), hydrogen cyanide (HCN), and tobacco-specific nitrosamines (TSNAs).

Nicotine, a pale yellow oily liquid with a characteristic tobacco odor, rapidly crosses the blood-brain barrier to stimulate dopamine release, inducing pleasure and serving as the primary basis for smoking addiction (Lewis, 2019). Chronic exposure compromises cardiovascular and respiratory systems, elevating the risks of heart disease, stroke, and lung cancer (Di et al., 2016). Furthermore, as a precursor, nicotine can transform into TSNAs under specific conditions, posing potential safety risks (Lewis et al., 2012).

PAHs are formed via the pyrolytic polymerization of macromolecular hydrocarbons (e.g., cellulose and lignin) under oxygen-deficient combustion conditions (McGrath et al., 2007). Tobacco smoke contains 16 toxic PAHs, including benzo [a]pyrene, a potent carcinogen linked to cancers, reproductive disorders, cardiovascular diseases, and obesity (Solomou et al., 2023). Furthermore, the thermal degradation of proteins and amino acids generates toxic gases such as HCN and quinoline. HCN is highly toxic, damaging the central nervous system, causing amblyopia, retrobulbar neuritis, and infertility, while also impairing wound healing in smokers (Oldham et al., 2014).

TSNAs are N-nitrosamines unique to tobacco products, formed mainly through the nitrosation of nitrogenous precursors like nicotine and nitrates (Lu et al., 2022). Possessing strong carcinogenicity, TSNAs are associated with various malignancies, including cancers of the oral cavity, nasal passages, esophagus, lung, liver, pancreas, and breast (Edwards et al., 2021). Consequently, mitigating these harmful chemical constituents in tobacco leaves remains a critical objective of tobacco fermentation.

### 2.2 Improving tobacco quality and flavor

Biotechnological interventions offer a strategic approach to modulate the coordination of internal chemical constituents in tobacco leaves. Specifically, these interventions aim to reduce the levels of undesirable components such as starch, proteins, cellulose, and nicotine, while simultaneously promoting the generation and accumulation of aroma-active compounds. This process ultimately enhances tobacco quality and smoking characteristics. Research indicates that the screening of functional microorganisms and enzymes, coupled with the optimization of fermentation conditions, can significantly shorten the aging cycle while improving the quality and aromatic profile of tobacco leaves (Yao et al., 2022; Mu et al., 2020; Xu et al., 2024).

In the realm of microbial fermentation for quality improvement, inoculating tobacco leaves with specifically screened functional strains can significantly accelerate the aging process and enhance sensory attributes. Early studies demonstrated that *Bacillus* and *Coccus* species isolated from tobacco leaves effectively improve leaf aroma. Recent research has further elucidated the synergistic effects of mixed microbial consortia; for instance, a specific ratio of *Bacillus amyloliquefaciens* to *Bacillus licheniformis* not only enhances aroma but also markedly reduces smoke irritation (Dai et al., 2020). Concurrently, species within the genus *Pseudomonas* exhibit potential for nicotine degradation, aiding in the modulation of physiological strength and toxicity reduction (Mu et al., 2020). Furthermore, enzymes, such as lipoxygenase and peroxidase, secreted during microbial metabolism catalyze the degradation of terpenes and higher fatty acids, generating key aroma-active compounds that reinforce the characteristic flavor profile of tobacco (Yao et al., 2022).

Regarding the application of enzyme preparations, the specific catalytic properties of exogenous enzymes enable precise macromolecular degradation. To address excessive starch content, the combined application of α-amylase and glucoamylase efficiently converts starch into water-soluble sugars, thereby enhancing sweetness and reducing off-notes (Xu et al., 2024). Conversely, to alleviate bitterness and irritation caused by proteins, the addition of neutral proteases and papain effectively degrades proteins, increasing amino acid levels and providing precursors for Maillard reactions (Zhang W. et al., 2023). To overcome the limitations of single-enzyme applications, recent research has prioritized the development and use of composite enzyme formulations. Studies indicate that scientifically blending amylases, proteases, cellulases, pectinases, and laccases enables the synergistic degradation of cell wall materials and intracellular macromolecules, reducing off-notes and improving aftertaste. Tobacco leaves treated with optimized composite enzyme formulations show reduced starch and protein contents alongside an increased total concentration of aroma-active compounds (Yang et al., 2014).

Moreover, to address the challenges of free enzyme instability and susceptibility to inactivation during processing, immobilized enzyme technologies and microencapsulation techniques are increasingly being adopted in tobacco quality enhancement. By immobilizing enzymes or microbes onto carrier materials, these methods not only extend the operational lifespan of biological agents and minimize environmental interference but also facilitate enzyme recovery and reuse, effectively lowering production costs (Liu et al., 2006). Consequently, biocatalytic technologies based on microbes and enzymes, operating by degrading undesirable components and generating aroma precursors, hold significant promise for the targeted improvement of tobacco quality and the deep enhancement of its flavor profile.

### 2.3 Utilization of tobacco by-products

**FIGURE 2:**
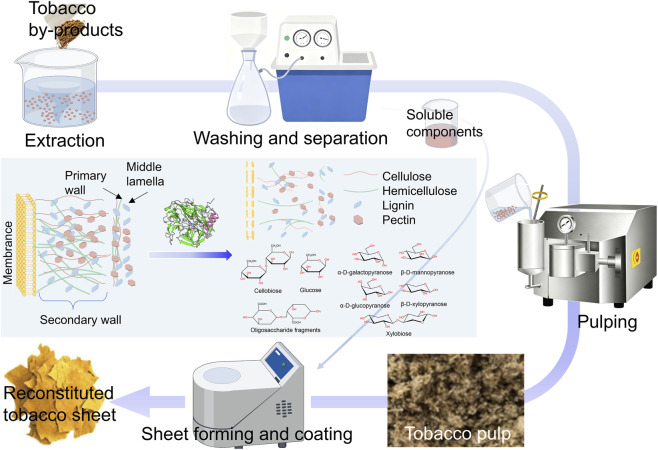
Process of reconstituted tobacco leaves.

Tobacco processing by-products constitute a collective residue from various stages of cigarette production, including tobacco stems, stalks, and leaves of inferior quality or with defects. Annually, a massive volume of such by-products is generated, representing a significant resource challenge (Jing et al., 2016). Achieving high-value utilization of these by-products is not only a practical necessity for mitigating environmental pollution but also a critical pathway for enhancing the economic efficiency of the tobacco industry. Currently, paper-making reconstituted tobacco sheet technology has emerged as the mainstream direction for resource utilization, owing to its superior physical properties, combustion characteristics, and tunability. This process converts low-value by-products into reconstituted tobacco leaves with performance comparable to natural cut filler through steps such as extraction, pulping, sheet forming, and coating. It effectively reduces cigarette production costs, improves product quality, and contributes to harm reduction (Luo et al., 2021) (Figure 2). However, the high content of lignin and cellulose in tobacco by-products leads to difficulties in pulping, while uncoordinated macromolecules such as proteins and pectins can induce sensory defects like heavy off-notes and strong irritation in the smoke, thereby constraining further improvements in product quality.

Microbial and enzymatic treatments can be applied across various stages of reconstituted tobacco sheet production. These biotechnological interventions exert positive effects on stem pulping efficiency, sheet base performance, and sensory quality, thus serving as key means to improve both processing performance and sensory attributes. During the raw material pretreatment and extraction stages, the application of cellulases, pectinases, and lignin-degrading enzymes can effectively disrupt the dense structure of stem cell walls. This not only increases the extraction rate of soluble and aroma-active components but also degrades macromolecules into Maillard reaction precursors, such as reducing sugars and amino acids (Bai et al., 2025). In the pulping process, enzymatic pretreatment softens the fiber structure, significantly reducing energy consumption during refining, improving fiber morphology, and consequently enhancing the physical strength and combustion properties of the sheet base (An et al., 2012). Furthermore, to address issues such as excessive nicotine content or insufficient aroma in by-products, biotechnology enables not only the targeted degradation of nicotine and removal of harmful substances like phenols but also promotes the synthesis of aroma-active compounds via microbial metabolic enzyme systems (Zhang W. et al., 2025).

## 3 Key enzyme systems and microbial community characteristics in tobacco biotransformation

### 3.1 Key enzymatic mechanisms underlying tobacco quality formation

**FIGURE 3:**
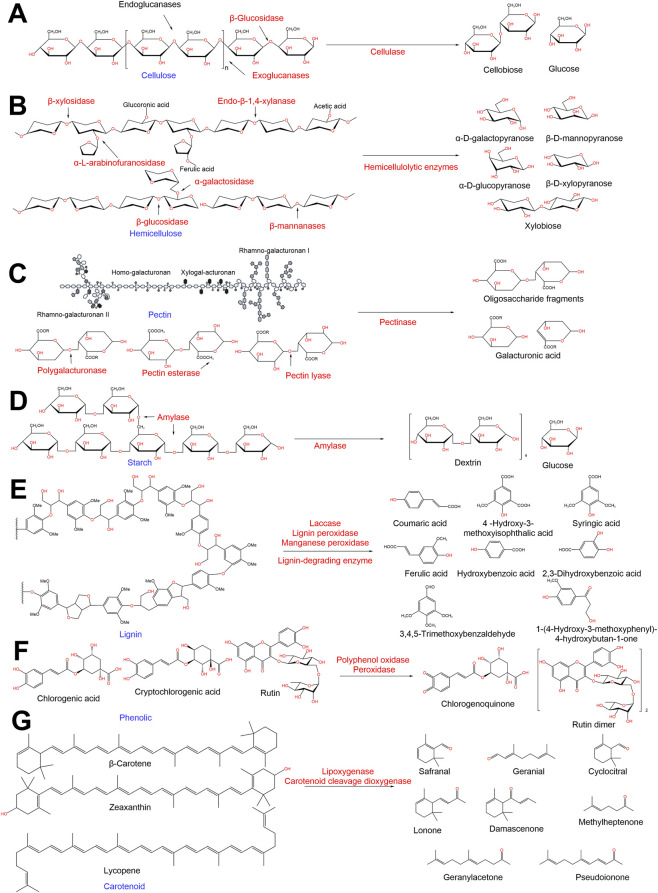
Enzymes in tobacco fermentation. **(A)** Cellulase; **(B)** Hemicellulolytic enzymes; **(C)** Pectinase; **(D)** Amylase; **(E)** Lignin-degrading enzyme; **(F)** Polyphenol oxidase and peroxidase; **(G)** Lipoxygenase and Carotenoid cleavage dioxygenase.

Enzymes serve as the pivotal biological drivers facilitating the degradation of macromolecules and their conversion into flavor constituents during tobacco aging. Enzymes with distinct functionalities can target specific leaf components, thereby eliciting diverse quality-enhancing effects (Figure 3).

#### 3.1.1 Carbohydrate-degrading enzyme system

Carbohydrates constitute the most abundant components in tobacco leaves. Existing forms of existence, content, and degree of degradation directly determine the physical structure, combustion characteristics, and accumulation of aroma precursors in the leaves (Han et al., 2025). The targeted degradation of macromolecular carbohydrates such as starch, cellulose, hemicellulose, and pectin represents the core element of biological enzyme-based quality improvement technologies.

The structural components of plant cell walls are complex polysaccharides, primarily consisting of cellulose, hemicellulose, and pectic substances. Cellulose is a homopolysaccharide composed of glucose units. To achieve complete hydrolysis of cellulosic material, the synergistic catalysis of endoglucanases, exoglucanases (cellobiohydrolases), and β-glucosidases is required to progressively depolymerize crystalline cellulose chains into cellobiose and ultimately hydrolyze them into glucose (Souza and Kawaguti, 2021). In tobacco processing, cellulases serve a dual purpose: physically, they disrupt the dense structure of cell walls and reduce cellulose crystallinity, thereby enhancing leaf flexibility and filling power while minimizing breakage during processing; chemically, the disintegration of the cell wall architecture breaks down mass transfer barriers, facilitating the release of intracellular aroma compounds. Furthermore, the resulting reducing sugars serve as high-quality substrates for Maillard reactions, providing the material basis for sweetness and fullness in the smoke. Hemicellulose, conversely, is a heteropolysaccharide composed of various hexoses and pentoses. Its efficient degradation requires the synergistic action of multiple hemicellulolytic enzymes, such as xylanases, mannanases, arabinofuranosidases, glucosidases, and xylosidases (Souza and Kawaguti, 2021). When acting in concert with cellulases, these enzymes further enhance the efficiency of cell wall degradation (Xu et al., 2024).

Pectin is a complex polysaccharide primarily composed of galacturonic acid. Its content is closely related to the cutting performance of tobacco leaves and the irritation level of combustion (Li et al., 2024). Through the synergistic catalysis of polygalacturonase, pectin esterase, and pectin lyase, the glycosidic linkages within pectin molecules are specifically cleaved, degrading high-molecular-weight pectin into galacturonic acid and oligosaccharidic fragments (Su et al., 2024). Studies indicate that pectinase treatment significantly disrupts the intercellular middle lamella structure, rendering tobacco leaf tissue loose and porous, thereby improving air permeability and combustion uniformity. More critically, the pyrolysis of pectin typically generates harmful substances such as methanol and formaldehyde, which are detrimental to human health and irritate the respiratory tract. Consequently, reducing pectin content via enzymatic hydrolysis represents a vital approach to lowering the release of harmful components and irritation in smoke from the source (Su et al., 2024; Weng et al., 2024).

During the aging process of tobacco leaves, starch degradation is a critical step, with α -amylase and glucoamylase serving as the key enzymes. Amylase rapidly cleaves the internal α-1,4-glycosidic bonds of starch molecules via an endo-acting mechanism, breaking down long-chain starch into short-chain dextrins and reducing system viscosity. Subsequently, glucoamylase sequentially removes glucose units from the non-reducing ends, achieving the efficient conversion of starch into water-soluble reducing sugars (Han et al., 2025). In the processes of tobacco mellowing or processing, the saccharification reaction driven by amylases not only optimizes the sugar-to-alkaloid ratio for better coordination but also provides abundant precursors for caramelization and Maillard reactions during subsequent curing or smoking. This plays a decisive role in enhancing the aroma and taste quality of tobacco (Wu et al., 2021).

#### 3.1.2 Enzyme system related to nitrogen metabolism

Enzyme systems related to nitrogen metabolism primarily act on nitrogen-containing compounds in tobacco leaves, with the core mechanism being the degradation of proteins by proteases. As the second most abundant class of macromolecules in tobacco leaves after carbohydrates, proteins, when present in excess, lead to harsh smoke, off-odors, and bitter aftertaste. Through a synergistic mechanism of endo- and exo-acting processes, proteases specifically cleave peptide bonds within protein molecules, gradually hydrolyzing them into polypeptides and free amino acids (Jiang C. et al., 2024). This enzymatic hydrolysis exerts a dual effect on improving tobacco quality: firstly, from the perspective of sensory safety, it directly reduces protein content, thereby minimizing the generation of harmful pyrolysis products at the source and alleviating the pungency and throat irritation of the smoke; secondly, from the perspective of flavor synthesis, the free amino acids produced by hydrolysis serve as crucial aroma precursors (Han et al., 2024). Research indicates that appropriate protease treatment can reduce protein content while increasing free amino acid levels in tobacco leaves. However, it is crucial to precisely control the degree of enzymatic hydrolysis to prevent the conversion of excess amino acids into undesirable flavors, ensuring the overall coordination of the final product quality (Zhang W. et al., 2023).

#### 3.1.3 Oxidoreductase system

The oxidoreductase enzyme system represents a complex catalytic network in the formation of tobacco quality, influencing leaf coloration, degrading harmful components, and generating characteristic aromas. Unlike hydrolases, oxidoreductases primarily drive the transformation and reconstruction of secondary metabolites—such as lignin, polyphenols, and pigments—through electron transfer and free radical reactions.

Lignin, serving as the filling matrix of the cell wall, possesses a complex aromatic structure that is difficult to degrade. Lignin-degrading enzymes, represented by laccase and lignin peroxidase, play a decisive role in this process. These enzymes utilize molecular oxygen as an electron acceptor to catalyze the oxidation of phenolic hydroxyl groups in lignin, disrupting its complex chemical bonds and leading to degradation or structural rearrangement (Song et al., 2019). This not only effectively reduces the woody odor and irritation of tobacco leaves but also removes the physical barrier of the cell wall, thereby enhancing the accessibility for subsequent enzymes such as cellulases (Su et al., 2016).

Oxidoreductases also play a core role in the color development of tobacco and targeted transformation of aroma precursors. Polyphenol oxidase and peroxidase can catalyze the oxidation of endogenous polyphenols into quinones and their polymers, which subsequently polymerize to form brown pigments, contributing to the desirable reddish-brown hue of cured tobacco (Zhang S. et al., 2025). Lipoxygenase catalyzes the oxidative degradation of unsaturated fatty acids, converting linoleic acid and linolenic acid into aldehydes and alcohols, which impart fresh green and fruity notes to tobacco (Jia et al., 2023a). Furthermore, acting in concert with carotenoid cleavage dioxygenases, these enzymes facilitate the oxidative degradation of carotenoids, generating key aroma components such as β-damascenone and β-ionone (Magome et al., 2023; Liang et al., 2021). Through catalyzing oxidation reactions, the oxidoreductase system achieves a dual objective: on one hand, it improves negative sensory experiences by degrading lignin and modifying polyphenols; on the other hand, it drives the degradation and metabolism of pigments and lipids to generate a rich array of small-molecule aroma compounds.

#### 3.1.4 Mechanism of compound enzyme systems

Tobacco fermentation constitutes a highly complex biochemical network, in which quality formation generally relies on coordinated metabolism of multiple components. In contrast to single-enzyme systems that only achieve targeted degradation toward specific substrates, compound enzyme systems can improve tobacco quality via cellular structure disruption, cascade catalysis and regulating microbial communities. First, tobacco cell walls are mainly composed of cellulose, hemicellulose and pectin, forming a dense physical barrier that impedes the catalysis activity of amylase, protease, lipoxygenase and other enzymes. Within the compound enzyme systems, pectinase and cellulase, and hemicellulase can effectively degrade the cell wall skeleton, thereby elevating the porosity and water permeability of tobacco leaf tissues (Souza and Kawaguti, 2021; Zhao et al., 2020). This structural modification not only accelerates the penetration and diffusion of enzyme molecules, but also provides abundant substrate binding sites for subsequent intracellular degrading enzymes such as proteases and amylases. Secondly, the synergistic effect of compound enzymes enables the directional transformation of aroma precursors through cascade catalysis. For instance, proteases catalyze the degradation of proteins to amino acids, while glucoamylase produces glucose, both of which facilitate the progression of the Maillard reaction (Hao et al., 2025). Studies have demonstrated that combined treatment with amylase and cellulase substantially alters total sugar profiles and reduces the contents of sugar, starch and cellulose in tobacco leaves, thereby improving the comprehensive quality of tobacco (Ning et al., 2023b). Ju et al. also reported that the combined application of cellulase or pectinase with protease markedly elevates the release efficiency of free amino acids in tobacco leaves, outperforming treatment with protease alone (Ju et al., 2023).

Furthermore, enzymatic treatment also modulates the structure and diversity of microbial communities during tobacco fermentation. Ning et al. revealed that amylase treatment induced distinct shifts in tobacco microbial community composition (Ning et al., 2023b). The rapid proliferation of *Bacillus*, *Sphingomonas*, and *Pseudomonas* triggered a reduction in bacterial diversity. This phenomenon can be attributed to the degradation of starch into reducing sugars by amylase, which greatly improves the bioavailability of carbon sources in tobacco leaves. Meanwhile, these strains are key contributors to nicotine degradation and the biosynthesis of characteristic flavor compounds (Zhang et al., 2020). Xu et al. reported that cellulase supplementation also substantially changed chemical composition and microbial composition (Xu et al., 2024). The relative abundance of *Aspergillus* decreased significantly, whereas the abundance of *Fibrobacter*, *Neurospora*, *Brevibacterium*, and *Komagataella* increased. During the fermentation process, the relative abundance of metabolism-related functional genes is elevated, and the expression of cellulases and peptidases also increased. The contents of starch, cellulose and total nitrogen in tobacco were ultimately reduced by 17.60%, 28.91% and 16.05%, respectively. Accordingly, the utilization of compound enzyme preparations has gradually evolved into a core strategy for modern biocatalysis in tobacco research.

### 3.2 Function of microbial communities during tobacco fermentation

The microbial community on the surface of tobacco leaves exhibits high diversity and complexity, with its composition primarily influenced by factors such as tobacco variety, origin, maturity, and curing methods. Research indicates that bacteria are the dominant group on the leaf surface, with abundance far exceeding that of fungi and actinobacteria (Wu et al., 2022). Major dominant genera include *Bacillus subtilis*, *Streptococcus*, *Lactococcus*, *Enterobacter*, *Pseudomonas*, and *Staphylococcus* (Wang et al., 2018). Among these, the genus *Bacillus* is almost present in all fermentation stages of tobacco leaves due to its tolerance to high temperatures and osmotic pressure, as well as its rich enzyme-producing capabilities, making it a core functional microbial group in tobacco fermentation. Although the fungal community is relatively less abundant, it plays an indispensable role in the transformation of flavor compounds (Di Giacomo et al., 2007; Jia et al., 2023b). Throughout the entire life cycle of tobacco, from growth and harvesting to curing and aging, these microorganisms form a unique micro-ecological network, providing the species foundation for subsequent fermentation and transformation processes. Notably, a single strain often cannot complete such complex transformation chains; instead, diverse microorganisms secrete a variety of enzymes. For example, co-culturing *B. amyloliquefaciens* with *Bacillus toyonensis* has been shown to yield higher levels of Maillard reaction products and terpenoid metabolites compared to other samples (Wu et al., 2021).

The aging process of flue-cured tobacco is a complex phenomenon involving microbial enzymatic actions and metabolic regulation. The appropriate addition of exogenous microorganisms can significantly improve tobacco quality (Table 1). Firstly, microorganisms serve as the primary biological source of enzymes. By secreting extracellular enzymes such as amylases, proteases, and cellulases, they decompose macromolecular substances in tobacco leaves, thereby increasing the accumulation of aroma compounds. Dai et al. isolated *B. subtilis* ZIM3, which can highly express the amylase AmyE1 and cellulase CelE1. After fermentation, the biodegradation efficiency of starch and cellulose in tobacco leaves increased by 30%–48% (Dai et al., 2020).

**TABLE 1:** Composition and mechanism of microbial preparations in tobacco fermentation.

Strains	Mechanism	References
*Bacillus subtilis* YCXW-01	Expressing cellulase, protease, amylase, xylanase	Yang et al. (2025)
*Bacillus velezensis* TB-1	Increasing volatile compounds	Shan et al. (2025)
*Bacillus amyloliquefaciens* ZH-2	Expressing proteases, chitinases, and β-1,3-glucanase Inhibits plant pathogens	Zhou et al. (2025)
*Bacillus subtilis* YY-10 *Bacillus subtilis* BY-2	Expressing lignin peroxidase and manganese peroxidase	Wang et al. (2025)
*Bacillus altitudinis*	Promoting nitrogen compounds degradation and modulating microbial metabolism to enhance aroma development	Liu et al. (2025)
*Bacillus halotolerans* NS36	Secreting enzyme systems associated with the degradation of lignin, cellulose, starch, and pectin	Wei et al. (2024)
*Bacillus mycoides* NS75	Secreting enzyme systems related to protein and glycoside hydrolysis, increasing Maillard reaction products and glycosylated compounds	Wei et al. (2024)
*Bacillus siamensis* DM-3	Expressing pectinase and increasing the diversity of both bacteria and fungi	Su et al. (2024)
*Bacillus subtilis* BSP1	Expressing protease and regulating the diversity of the microbial community on the surface of the tobacco leaves and the ubiquitin-proteasome pathway	Jiang F. et al. (2024)
*Bacillus subtilis* BS3	Regulating the activities of peroxidase, superoxide dismutase, polyphenol oxidase, starch branching enzyme and amylase	Zhang Y. et al. (2024)
*Bacillus amyloliquefaciens* W6-2	Expressing pectinase and regulating bacterial communitie	Weng et al. (2024)
*Bacillus velezensis A2* *Bacillus endophyticus* A4	Expressing amylase, cellulase, and protease and increase the abundance of *Bacillus*, *Pseudomonas* and *Klebsiella*	Zhang L.-Y. et al. (2024)
*Bacillus subtilis XP01*	Expressing protease and amylase	Ma et al. (2024)
*Bacillus velezensis* HJ-16	Expressing cellulase, protease, and amylase, reducing diversity and evenness, and enhancing microbial metabolic activity	Zhou et al. (2024)
*Pseudomonas stutzeri* ZCJ	Promoting the degradation of nicotine	Zhao et al. (2012)
*Candida parapsilosis* P1	Promoting the degradation of alkaloids, chlorophyll, and carotenoids	Jia et al. (2023a)
*Bacillus sp. YC7*	Expressing nicotine dehydrogenase, β-glucosidase, cellulase, proteases, and amylases	Zhang K. et al. (2023)
*Bacillus amyloliquefaciens LB* *Bacillus kochii SC*	Expressing protease and amylase	Wu et al. (2021)
*Bacillus amyloliquefaciens* B9601-Y2	Showing antifungal activity and inhibiting the mycelial growth of fungal pathogen	Pan et al. (2021)
*Bacillus subtilis* ZIM3	Expressing amylase and cellulase	Dai et al. (2020)
*Bacillus subtilis Tpb55*	Increased the activity of peroxidase, catalase, superoxide dismutase, and β-1,3-glucanase, and inhibiting the growth of *Phytophthora nicotianae*	Zha et al. (2017)
*Myceliophthora thermophila*	Expressing pectinase MtPly-1, MtPly-2, and NcPly-2	Zhang M. et al. (2025)
*Phaffia rhodozyma*	Synthesizing carotenoid-astaxanthin and regulating bacterial communitie	Mai et al. (2024)
*Filobasidium magnum*	Expressing lipid oxidase	Wen et al. (2023)
*Saccharomyces cerevisiae* *Clavispora lusitaniae* *ZygoSaccharomyces rouxii* *Saccharomycopsis fibuligera* *Hanseniaspora uvarum* *Pichia pastoris* *Cyberlindnera fabianii*	Synthesizing volatile substances such as alcohols, ketones, esters, aldehydes and neophytadiene	Yao et al. (2022)
*Phanerochaete chrysosporium* *Trametes hirsute* *Trametes versicolor*	Expressing lignin peroxidase, manganese peroxidase, laccase, endoglucanase, exoglucanase and filter paper enzyme. Promoting the degradation of nicotine	Su et al. (2016)

Secondly, the unique metabolic pathways of microorganisms directly enrich tobacco aroma components and reduce harmful substance levels. Microorganisms not only convert precursors into aroma compounds but also synthesize flavor substances directly through secondary metabolism. Yao et al. selected nine strains of aroma-producing yeast with excellent liquid fermentation performance for artificial solid-state fermentation, and found 52 aroma compounds contributed to the flavor of cigar in all treatment groups (Yao et al., 2022). Certain functional strains can synthesize carotenoids and degrade them into key aroma components like β-damascenone and megastigmatrienone (Mai et al., 2024). Furthermore, microorganisms like *Pseudomonas*, *Arthrobacter,* and *Cunninghamella echinulata* degrade excess nicotine into non-toxic or less irritating substances via the pyrrolidine, pyridine, and mevalonate pathways (Mu et al., 2020; Li et al., 2024). Additionally, microbial succession can inhibit the growth of harmful molds through competitive exclusion, thereby reducing the risk of tobacco mildew and minimizing the accumulation of harmful precursors like TSNAs (Zhou et al., 2025; Pan et al., 2021).

## 4 Discovery, engineering, and mechanistic validation of biological resources for tobacco quality improvement

Enzymatic treatment and inoculation serve as effective strategies for regulating the chemical composition, enriching the flavor profile, and enhancing the sensory quality of tobacco, thereby exhibiting broad application prospects. However, natural enzyme preparations often suffer from low catalytic activity and poor stability. Furthermore, traditional screening approaches cannot efficiently isolate high-efficiency functional strains. These limitations severely constrain the full realization of biotechnological potential in tobacco processing. Previous research mainly focused on the application of commercial enzyme preparations and conventional strain screening, while failed to deeply explore superior biological resources with specific adaptability to the tobacco matrix. Consequently, this chapter systematically elaborates on the discovery and rational design of high-efficiency enzymatic resources, as well as the mining of functional microbial resources for tobacco processing.

### 4.1 Mining and rational design of enzyme resources

**FIGURE 4:**
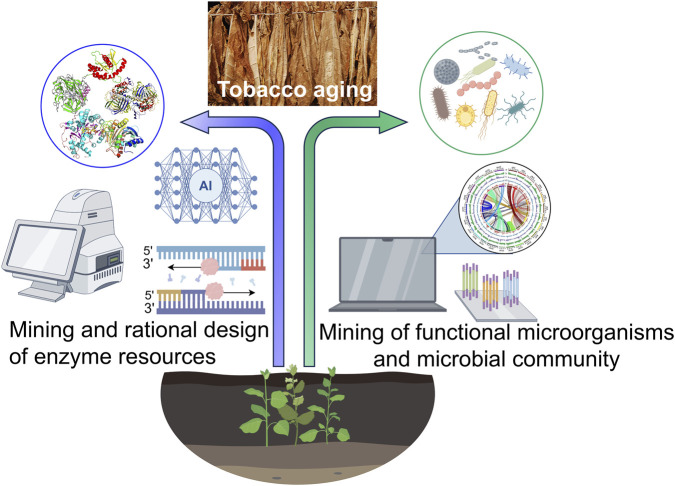
Discovery and Engineering of biological resources.

Traditional enzyme mining has primarily relied on screening high-yielding strains from natural sources. However, the enzymatic properties often fail to meet the demands of the high-temperature and alkaline conditions inherent to tobacco fermentation. The maturation of genetic and protein engineering technologies provides a robust technical foundation for acquiring industrial-grade enzymes (Figure 4).

The tobacco leaf surface and fermentation environment harbor a vast reservoir of untapped microbial genetic resources. Mining novel enzyme genes from these environmental metagenomes represents a potent strategy to overcome the inherent limitations of natural enzyme preparations. For instance, Li et al. screened a pectin lyase-producing strain from tobacco leaves and identified a novel pectin lyase Pel04. This enzyme demonstrated stability within the range of 30 °C–50 °C, and under alkaline conditions (pH 9.5–11), indicating significant potential for tobacco quality improvement (Li and Tian, 2024).

To address the low catalytic efficiency of native enzymes within the complex tobacco matrix, protein engineering offers a precise and robust solution. Through strategies such as site-directed mutagenesis, error-prone PCR, and gene shuffling, it is possible to systematically optimize the active site and tertiary structure of enzymes. Deay et al. successfully enhanced the oxidative turnover rate of the nicotine oxidase NicA2 enzyme by 25% and reduced the *K*_m_ of the NctB protein by 34% through site-directed mutagenesis (Deay et al., 2022). Notably, artificial intelligence-guided protein design has recently emerged as a transformative approach. In a previous study, the deep learning model ProteinMPNN was employed to comprehensively redesign the sequence of a pectinase, guided by multiple sequence alignment. The variant DS-5 with 72 mutations exhibited an 8.9-fold increase in catalytic activity, a 10 °C increase in optimal reaction temperature, and remarkable stability across a broad pH range (7.0–11.0). Consequently, the sensory profile of tobacco treated with DS-5 was improved through the elevation of desirable flavor compounds such as sucrose and lactones (Zheng et al., 2026). These studies demonstrate the value of rational design in developing high-efficiency enzymatic preparations tailored for tobacco applications.

### 4.2 Mining of functional microorganisms and microbial community construction

Microorganisms serve not only as the executors of tobacco fermentation but also as natural factories for the production of extracellular enzymes. Isolating core functional strains from natural fermentation systems and constructing artificial synthetic microbial communities based on ecological principles represent a key pathway for the targeted improvement of tobacco quality (Figure 4).

The integration of culture-dependent isolation with multi-omics analysis has improved the efficiency and accuracy of functional microorganism discovery. Traditional screening approaches usually depend on pure culture isolation and single-function assays, such as protease, amylase, cellulase, or nicotine-degradation activity. Although useful, these methods may overlook uncultured microorganisms or strains that are functionally important only within complex communities. By contrast, metagenomics, metatranscriptomics, and metabolomics can link microbial composition with functional genes, active metabolic pathways, and chemical changes during tobacco aging. For example, Zhang et al. analyzed microbial and enzymatic dynamics during cigar tobacco air-curing and fermentation and found that bacterial communities were mainly associated with sugar, amino acid, and lipid metabolism, whereas fungal communities contributed to lignin, cellulose, and pectin transformation through saprotrophic activity (Zhang M. et al., 2025). These findings indicate that bacteria and fungi may play complementary roles in tobacco matrix remodeling.

Beyond identifying individual functional strains, future research should focus on the rational construction of synthetic microbial communities. Natural tobacco aging involves complex interactions among bacteria, fungi, enzymes, and plant-derived substrates. A single strain is unlikely to reproduce the full range of biochemical transformations required for balanced flavor formation. Synthetic consortia can be designed by combining strains with complementary functions, such as protease-producing bacteria for protein degradation, amylase- or cellulase-producing strains for carbohydrate conversion, oxidoreductase-producing fungi for phenolic and terpenoid remodeling, and nicotine-transforming bacteria for alkaloid regulation. However, the construction of such communities should be guided by metabolic compatibility, ecological stability, colonization ability, and performance under low-moisture solid-state conditions rather than by simple strain mixing.

### 4.3 Multi-omics strategies for validating microbial–enzymatic mechanisms

A major limitation of current studies is that microbial or enzymatic interventions in tobacco processing are often evaluated mainly through endpoint chemical measurements, enzyme activity assays, or sensory scores. Although these indicators are useful for assessing quality changes, they provide limited information on the causal mechanisms linking microbial communities, enzyme systems, substrate conversion, metabolite formation, and sensory improvement. This limitation is particularly important for tobacco aging, which occurs in a heterogeneous solid-state matrix characterized by low moisture, uneven oxygen diffusion, spatially variable nutrient availability, and restricted mass transfer. Under such conditions, microbial abundance does not necessarily indicate metabolic activity, and enzyme activity measured in liquid assays may not represent catalytic performance in the tobacco matrix. Therefore, multi-omics strategies are required to move the field from descriptive association toward mechanism-oriented validation.

Metagenomics can reveal the taxonomic composition and functional gene potential of tobacco-associated microbial communities, including genes encoding proteases, amylases, cellulases, pectinases, oxidoreductases, glycosidases, nicotine-degrading enzymes, and nitrate reductases. However, the presence of these genes does not prove that they are active during aging. Metatranscriptomics can identify genes that are transcriptionally activated under specific processing conditions, whereas metaproteomics can provide more direct evidence of enzymes actually produced in the tobacco matrix. When combined with targeted enzyme activity assays, these approaches can help verify whether specific enzyme systems are functionally involved in protein hydrolysis, starch saccharification, cell-wall degradation, phenolic oxidation, terpenoid remodeling, or alkaloid transformation. Metabolomics provides the chemical endpoint of microbial–enzymatic transformation by tracking the depletion of substrates and the accumulation of products, including sugars, amino acids, organic acids, phenolics, terpenoids, alkaloids, fatty acid derivatives, and volatile aroma compounds. When integrated with sensory evaluation and smoke chemistry, metabolomics can link biochemical changes to aroma intensity, sweetness, smoothness, irritation, aftertaste, off-odor reduction, and safety-related indicators such as nicotine, nitrate, tobacco-specific nitrosamines, aldehydes, and polycyclic aromatic hydrocarbon-related precursors. This integrated evaluation is necessary because microbial–enzymatic treatments may improve flavor attributes while also altering the formation potential of undesirable compounds.

To strengthen causal inference, multi-omics analysis should be combined with time-series sampling, perturbation experiments, isotope-assisted substrate tracing, and predictive modeling. Correlation networks, co-occurrence analysis, sparse partial least squares regression, two-way orthogonal partial least squares, random forest, and structural equation modeling can be used to connect microbial taxa, functional genes, enzyme abundance, metabolite dynamics, and sensory traits. However, statistical associations should be further validated through microbial inoculation, enzyme addition, strain removal, inhibitor treatment, or labeled-substrate tracing. For example, ^13^C-labeled starch, amino acids, linoleic acid, or carotenoid derivatives could be used to trace carbon flow from specific tobacco precursors into flavor-related metabolites, thereby distinguishing active functional contributors from passive community members.

Overall, multi-omics strategies should be regarded not only as descriptive profiling tools but also as platforms for mechanism validation and process prediction. A robust evidence chain should link microbial community structure, functional gene potential, enzyme expression, substrate conversion, metabolite formation, sensory quality, and safety-related responses. Such a framework would provide a stronger scientific basis for the rational development of functional strains, synthetic microbial consortia, engineered enzymes, and precision enzyme cocktails for controllable tobacco aging and by-product valorization.

## 5 Conclusion

The integration of microbial fermentation and enzymatic engineering is poised to transform tobacco processing from an empirical, time-consuming art into a precise, controllable biotechnological science. This review has systematically elucidated the critical roles of key enzyme systems and dynamic microbial communities in modulating tobacco’s chemical matrix. By facilitating macromolecular degradation and facilitating the biosynthesis of aroma precursors, these biological agents significantly enhance sensory quality, improve combustion characteristics, and mitigate harmful constituents such as TSNAs and nicotine.

Furthermore, the application of metagenomics has unlocked a vast reservoir of novel enzymes adapted to the tobacco niche. Concurrently, protein engineering, particularly AI-guided design, has enabled the creation of enzyme exhibiting superior stability and catalytic efficiency under industrial conditions. In conclusion, the convergence of enzymology, microbiology, and computational biology holds immense promise for the next-generation of tobacco manufacturing. By utilizing these advanced biotechnological tools, the industry can achieve a delicate balance between preserving traditional flavor profiles and meeting modern demands for reduced harm and enhanced consistency, ultimately driving a sustainable and high-quality future for tobacco products.
